# Reply to van Bruggen, F.H.; Luijendijk, H.J. Comment on “Chwal et al. On-Target Low-Density Lipoprotein Cholesterol in Adults with Diabetes Not at High Cardiovascular Disease Risk Predicts Greater Mortality, Independent of Early Deaths or Frailty. *J. Clin. Med.* 2024, *13*, 7667”

**DOI:** 10.3390/jcm14082845

**Published:** 2025-04-21

**Authors:** Bruna C. Chwal, Rodrigo C. P. d. Reis, Maria I. Schmidt, Antonio L. P. Ribeiro, Sandhi M. Barreto, Rosane H. Griep, Paulo A. Lotufo, Bruce B. Duncan

**Affiliations:** 1 Programa de Pós-Graduação em Epidemiologia, Universidade Federal do Rio Grande do Sul, Porto Alegre 90035-003, Brazil; brunacristine.chwal@gmail.com (B.C.C.); rodrigocpdosreis@gmail.com (R.C.P.d.R.); maria.schmidt@ufrgs.br (M.I.S.); 2 Departamento de Estatística, Universidade Federal do Rio Grande do Sul, Porto Alegre 91509-900, Brazil; 3 Hospital de Clínicas de Porto Alegre, Porto Alegre 90035-903, Brazil; 4 Faculdade de Medicina e Hospital das Clínicas/EBSERH, Universidade Federal de Minas Gerais (UFMG), Belo Horizonte 30130-100, Brazil; antonio.ribeiro@ebserh.gov.br (A.L.P.R.); sandhi.barreto@gmail.com (S.M.B.); 5 Laboratório de Educação em Ambiente e Saúde, Instituto Oswaldo Cruz, Fundação Oswaldo Cruz, Rio de Janeiro 21040-900, Brazil; rohgriep@gmail.com; 6 Center for Clinical and Epidemiologic Research, University of São Paulo, Sao Paulo 05508-000, Brazil; palotufo@usp.br

We thank Drs. van Bruggen and Luijendijk for their insightful comments [1], bringing yet another all-cause mortality study, this one being a randomized trial, questioning the generally accepted notion in the care of diabetes: the lower the LDL-C, the better. The increased (HR 1.10; 95% CI 0.91–1.32) mortality among those with diabetes when treated for very low LDL-C levels as highlighted in the FOURIER trial, though not statistically significant, is concerning, especially within the context that most trials of intensive lipid-lowering in diabetes have not reported all-cause mortality.

No meta-analysis of trial data shows that lowering LDL-C beyond 100 mg/dL in patients with diabetes without pre-existing, clinically evident atherosclerotic cardiovascular disease produces any benefits when considering all causes [2,3]. Until this benefit is documented, the currently limited trial evidence to support more intensive lipid-lowering coupled is insufficient. These trials, coupled with multiple observational studies that, though subject to risk of bias and reverse causality, find harm with lower levels of LDL-C in diabetes, should constitute a red flag for those making guidelines. Clinical trials of intensive lipid-lowering in diabetes reporting all-cause endpoints, including mortality, are much needed.

Approximately 10% of the world’s adults are estimated to have diabetes [4]. Recent research suggests that most of them will no longer die from cardiovascular diseases [5]. In fact, in England, only 24% died of ischemic heart disease in 2018 [6]. Despite the limited evidence of all-cause benefits, lipid-lowering treatment, which has become increasingly intensive over time, is a near-universal recommendation for most with diabetes. Thus, this issue is of major importance to public health.

The role of low LDL-C levels as a risk for cancer and in vulnerability to infectious complications in diabetes appear to be central questions for investigation. Further Mendelian randomization studies in this area are important. We, in the ELSA-Brasil study, intend to provide further input on these issues as our outcome data accumulates. We hope other researchers will also investigate this issue, as it directly impacts the clinical care of tens of millions of patients worldwide.
